# Exploratory pilot study on resource allocation along the dementia continuum under constrained and unconstrained budget scenarios

**DOI:** 10.1186/s12877-022-03089-1

**Published:** 2022-05-18

**Authors:** Tom Pierse, Fiona Keogh, David Challis, Eamon O’Shea

**Affiliations:** 1 Centre for Economic and Social Research on Dementia, National University of Ireland Galway, Galway, Ireland; 2 National Doctors Training and Planning, Health Service Executive, Dublin, Ireland; 3 Mental Health Ireland, Dublin, Ireland; 4 University of Nottingham Innovation Park, Institute of Mental Health, Nottingham, UK

**Keywords:** Dementia, Balance of care, Longitudinal, Resource allocation

## Abstract

**Background:**

People with dementia and their carers have a wide range of health and social care needs which vary along the dementia continuum. The government response to events and transitions at various stages of the continuum can have a substantial impact on the lived experience of dementia and to resource allocation decision-making. Hearing what practitioners have to say about need at various points of transition along the dementia continuum is very important, especially for the resource allocation process.

**Methods:**

The paper uses an innovative longitudinal balance of care (BoC) methodology to identify the impact of changes along the dementia care continuum for care recipients and practitioners throughout the course of the condition. Participatory workshops were held with five Health and Social Care Professionals (HSCPs) to pilot a mixed methods approach to resource allocation decision-making along the dementia continuum. In these workshops, these practitioner participants were asked to generate a set of services and supports for a person with dementia with changing and evolving needs over a five year period under two budget scenarios: no budget constraint (NBC); and a budget constraint (BC). Participants were asked to recommend services for short, post event, transition periods and for longer steady state periods.

**Results:**

Participants were able to allocate different packages of services and supports for different stages of dementia under different budgetary conditions. The total cost for the five year period under the NBC scenario is €200,000 on average, reducing to €133,000 under the BC scenario. Under the BC (NBC) scenarios, participants spent on average 85% (90%) of their budget on community services and 15% (10%) on nursing home services.

**Conclusion:**

The methodology used in this paper is a valuable complement to cross-sectional BoC studies through its identification of the importance of events, transitions and staging along the dementia care continuum. The desire of participants to keep people with dementia living in their own home is strong, even in the later stages of dementia, as evident by their recommendation to spend €400 per week more on home care provision compared to the alternative residential care, albeit in the absence of any budget constraints.

**Supplementary Information:**

The online version contains supplementary material available at 10.1186/s12877-022-03089-1.

## Introduction

Dementia is a loss of cognitive functioning that impacts on memory, language, mood, personality, and the ability to carry out everyday activities [1]. Among older people, dementia is the most common cause of functional and cognitive decline [2]. People with dementia and their carers have, therefore, a wide range of health and social care needs requiring attention from government [3]. These needs vary across the dementia continuum. For people with mild symptoms of dementia, key needs include a confirmed diagnosis, information and emotional support [4]. As the condition progresses, assistance with social activities, household tasks or personal care may be required. While most people want to be cared for at home for as long as possible [5], at some point, it may not be possible to continue caring for the person at home and a move to a nursing home is the only available option [6]. Most countries try to postpone admission to nursing homes for as long as possible and practicable [7–9], and a range of community-based interventions have been developed, some more cost effective than others [10, 11] and a few that seek to reflect the preferences of people with dementia and their carers [12, 13].

Community services for people with dementia in Ireland are mainly funded by government and delivered through a combination of public, voluntary and private providers [14]. There are substantial disparities across the country in the type and availability of community services [15]. Resource allocation is rooted in incrementalism and a historical approach to priority-setting within social care provision in Ireland [16]. Home care is typically limited to public health nursing (providing health promotion, health protection and community-based clinical nursing care) and home help services, sometime extending to broader psychosocial supports, depending on where people live. More intensive home care support programmes, the focus of this study, have received increasing attention in recent years resulting from the publication of the Irish National Dementia Strategy and additional resources being made available by government.

With aging populations driving increased demand [17], and limited budgets, a key challenge for policy makers is to identify how resources can be allocated efficiently along the dementia continuum. There are limitations to the current methods used to prioritise services for people with dementia. Met and unmet needs can be directly surveyed [18], but, to be useful, these have to be translated into a set of costed service recommendations for resource allocation decision making. Discrete choice experiments [12, 13] are sometimes used to rank service attributes and levels, including the public’s willingness to pay additional taxation in support of them, but are not always suited to allocating resources at a micro level, especially at key transition points along the continuum. Similarly, cost effectiveness analysis of a particular service for people with dementia [19] is not always suited to complex resource allocation decision-making.

The Balance of Care (BoC) method have been used more generally to identify the service needs of older people on the margin of home/community and residential care. It has also been used to identify how stakeholders allocate resources across representative case types of dementia with different needs. Balance of care studies have four defining characteristics: identification and measurement of client characteristics that affect decisions about the most appropriate setting in which to support them; the specification of available resources; some means of allocating clients to the most appropriate setting; and a determination of the costs of care in different settings [20]. Within BoC, decision-making is usually presented in static terms for participants requiring them to make judgements on care needs and subsequent resource allocation [21]. Most BoC studies are, therefore, cross-sectional – they use a small number of distinct case types described by a set of key characteristics specific to both time and place.

In reality, stakeholders are aware of potential changes in resource allocation for people with dementia over the course of the disease. It is not only that people have different needs at different times, but a decision to invest early in the trajectory of the disease may have consequences for what happens subsequently. For example, additional services for people living at home earlier in the disease may delay the potential transition into long-stay care, sometimes avoiding it altogether [22]. Dealing with foreseen and unforeseen events are also a key part of care planning for people with dementia [23]. The quality of care around critical events, such as diagnosis or hospitalisation, can have a significant impact on the lived experience of people with dementia [4] and outcomes such as re-hospitalisation [24, 25]. Diagnosis may activate post diagnostic supports, community mental health team support and reablement, but some of these services may only be typically provided for a limited duration following an event [4, 26]. Similarly, home support hours and intensive home care packages are frequently allocated around admission and discharge from acute care [27, 28].

It is important, therefore, to consider resource allocation decision-making around key events and transitions. Decision-makers must balance current needs against future needs and create optimal pathways of care that balance the desire of people to remain living at home for as long as possible and practicable with associated risks and costs. In this pilot study we outline an approach, within the BoC methodology, to generate information on resource allocation for a person with dementia along a typical pathway of care, under unlimited and limited budget scenarios. We ask participants in the BoC exercise to identify stage appropriate services and supports for a typical person with dementia over a period of five years. The stages are: mild stage; two moderate stages incorporating enhanced functional impairment and behavioural symptoms; and a severe stage where the person with dementia is fully dependent. Transition events are identified as the person with dementia moves along the continuum.

## Methods

**Fig. 1:**
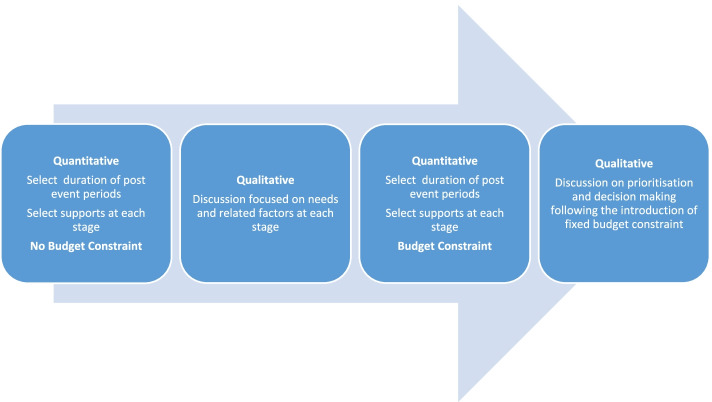
Research design

This study builds on a previous study that used a cross-section or ‘point in time’ BoC methodology with six case types of dementia [21, 29, 30]. In the course of this previous study, it became apparent that the ‘point in time’ approach did not adequately address the progressive and deteriorating nature of dementia and associated decision making regarding service provision. The data reported here is a pilot study with the novel application and addition of a longitudinal case type to the BoC methodology, which may have relevance to resource allocation decision-making across many countries, outside of Ireland. An explanatory sequential design was used in this study, involving an initial quantitative phase, followed by allocation exercises and associated qualitative discussion with the participants, as shown in Fig. 1 [31]. A Nominal Group Method (NGM) was used within an overall BoC methodology to capture both qualitative and process data on the approach to decision-making [30]. This is a pilot study intended to support the conduct of a much larger study in the future [32].

There was a strong commitment to Pubic Patient Involvement (PPI) in this study. PPI is defined as research carried out ‘with’ or ‘by’ members of the public, rather than ‘to’, ‘about’ or ‘for’ them; the term ‘public’ includes patients, potential patients, carers and people who use health and social care services, as well as organisations that represent people who use these services [33, 34]. PPI facilitates current and future patients having a greater role in research prioritisation and research design, which is important given the potential of health research in finding solutions to their specific health needs [35]. The list of services was developed in consultation with two people with dementia and two carers and modifications were made to both as a result. A person with dementia was a member of the Oversight Group for the overall study.

### Participants

A convenience sample of five Health and Social Care Professionals (HSCPs) participated in this pilot study. Three social workers from a hospital-based team were involved, alongside a home care coordinator and an occupational therapist. Hospital based social workers see both the hospital and community perspectives on care and tend to have a broader non-medical focus. Home care coordinators have a key resource allocation role currently in the Irish system, given that home care is the largest community-based service for people with dementia. The occupational therapist is an important reablement support and serves as a referral pathway for other community services. Two workshops with participants were carried out in two different regions of the country. Both workshops were recorded with the permission of participants.

### Services

The service list provided to participants was informed by a mapping study of dementia-specific services in Ireland carried out in 2018 [36]. The list shown in Table 1 contained a variety of bio-psychosocial supports and was previously used in a conventional BoC exercise for people with dementia in Ireland [30]. Twenty three community-based supports and nursing home placement were included on the list presented to participants. Although each of these services is available in some parts of Ireland, most are not universally available and therefore may not be familiar to some people. The services were categorised into functions such as basic provision (public health nursing, home help, day care, meals on wheels), community health, respite and social/getting out to make the exercise easier to complete. A short description of each of the services was provided to participants, so that they would have full information when making allocation decisions. The study was structured specifically to encourage people to think beyond the usual set of services provided. This was done by starting the exercise without a budget constraint and having a broad range of services available. While there are potentially more services that could have been included on the service list, the number was limited to twenty three so that the exercise did not become overly complex.

**Table 1:** Service list

Basic services and supports	
Home Help	
Day Care (Standard or Dementia Specific)	
Meals on Wheels	
Public Health Nurse	
**Community Health**	
Physio	
OT	
Other Primary Care (Speech and Language/Dietician/Hearing)	
Referral to Psychiatry of Old Age Team	
Day Hospital (Primary Care Centre)	
Dementia Advisor	
Specialist Dementia/Case Management	
Dementia Cognitive Therapies	
Counselling for Person with Dementia	
Social Worker	
**Respite**	
In-home Respite/Sitting Service (eg visiting service)	
Nursing Home based Respite	
**Carer Services**	
Carer Education Programme	
Dementia Carer Support Groups	
Counselling for Family Carer	
**Social/getting out and about**	
Transport	
Re-ablement / Dementia Support Worker	
Alzheimer’s Café, Dementia Social Clubs, or other support group for people with dementia	
Dementia Friendly Activities	
**Nursing Home Bed**	

### Time period

Population-based studies indicate that people aged 65 years or older live for on average three to nine years after a diagnosis of dementia [37]. A Swedish study, using DSM-III-R criteria, showed newly diagnosed cases in people over 75 years of age spent 2 years in the mild phase, 1–2 years in the moderate stage, and a year in the severe stage [38]. Based on these trajectories, we choose a five year trajectory for the representative person with dementia in our study. We divided the moderate stage into two time periods to allow for the possibility that sometimes, across this stage of the care continuum, social care needs can increase for physical ADL reasons, for example, dressing and bathing, and behavioural reasons, for example, hallucinations.

This means that participants were asked to consider four time periods over the five years, each consisting of 65 weeks (260 weeks in total). The Mini-Mental State Examination (MMSE) was used to determine the severity of dementia [38]. Cut points on the MMSE of 9 and 17 were used to define and differentiate severe, moderate and mild dementia respectively. These broad stages are linked with a range of characteristics, as shown in Table 2.

**Table 2:** Dementia trajectory showing the changing situation over time

	Post Event	Steady State	Post Event	Steady State	Post Event	Steady State	Event	Steady State
**Characteristics**	**Period 1a**	**Period 1b**	**Period 2a**	**Period 2b**	**Period 3a**	**Period 3b**	**Period 4a**	**Period 4b**
**Event at start of period**	**Diagnosis.**		**Increased care needs. Some degree of supervision necessary.**		**Hospitalised eg. minor fall, UTI, pneumonia.**		**Increased care needs and carer strain. Trigger event.**	**Nursing Home.**
Dementia Severity	Mild		Moderate		Moderate		Severe	
Functional Impairment	None		Shopping, Transport		Dressing, Bathing		Fully Dependent	
Behavioural Symptoms	Apathy		Apathy		Apathy, Hallucinations		Apathy, Hallucinations	
Comorbidities	Diabetes		Diabetes		Diabetes		Diabetes & swallowing problems	
Living Situation	Spouse (75) + children near		Spouse (77) + children near		Spouse (78) + children near		Spouse(79) + children near	
Carer Burden	Low		Moderate		Moderate		High	
Length of period in weeks	**0**	65	**0**	65	**0**	65	**0**	65
Total No. of Weeks per period		65		65		65		65

### Characteristics of the representative person with dementia

In line with previous BoC studies, five characteristics of the representative person with dementia were chosen to vary over time: functional impairment, behavioural symptoms, comorbidities, living situation and carer burden [21]. Functional impairment was varied from none to fully dependent across the four stages [39]. Apathy and hallucinations were chosen as behavioural symptoms, the latter a proxy indicator for increasing dependency over the moderate stage of the continuum [40, 41]. Diabetes is included as a co-morbidity due to the frequency with which related medication use occurs in people with dementia [40, 41]. The person with dementia was assumed to be supported by their spouse and adult children [42, 43]. Carer burden was adjusted from low in the early stage to moderate in the two intermediate stages and high in the later stage, due to increases in behavioural symptoms, enhanced care needs and crises management [44, 45].

### Transitions

Five events were chosen as transition points across the various stages: initial diagnosis; changes in care needs; hospitalisation; enhanced carer burden; and potential for nursing home admission. Diagnosis is a key entry point to the dementia resource continuum. In this study, it is assumed that the diagnosis happens early, but it is acknowledged that, in reality, this is not always the case [46]. However, diagnosis is often the catalyst for services and support to be considered and activated. The next transition is marked by an increase in need as the person requires assistance with some tasks, reflecting an increase in both physical and social constraints. A hospitalisation event was chosen as the next transition point, as this is when more intensive and consistent home care may be needed. As care needs expand so too does the potential for an increase in carer burden. The latter can be activated through an unforeseen trigger event, such as carer sickness/hospitalisation or behavioural changes in the person with dementia [45, 47, 48]. Ultimately, the ability of the person with dementia to continue to live at home becomes an issue for the formal care system and for family carers, raising the potential of their admission into long-stay residential care.

### HSCP workshop

Each workshop participant was given a computer with a spread sheet workbook pre-loaded. The first tab of the workbook is shown in Table 2. This tab shows the characteristics of the typical person with dementia over time. Each period is split in two parts – “post event” and “steady state”. Post event is the short period directly after a transition point; steady state is the longer period when care provision has adjusted to the initial transition shock. Participants are invited to vary the length of the post event period which automatically changes the length of the steady state period. The other tabs of the workbook showed the list of services that could be allocated to the typical person with dementia for each of the sub-periods. Unit costs were embedded in the workbook, but were hidden initially during the loosening of the budget constraint. The cost of a nursing home bed is based on an average weekly charge for private and voluntary nursing homes on the Nursing home Support Scheme 2018 [49].

The first exercise was the No Budget Constraint (NBC) scenario. Participants were asked to allocate the amount of services that would be of most benefit to the person with dementia and their carers in each time-period. Each participant was asked to discuss and reflect on their thought process in putting together the care package at different time points and the needs that they were trying to address.

The second exercise involved decision-making under a Budget Constraint (BC) scenario, working within an overall budget constraint of €100,000. This level was set with reference to two years of high intensity home-based care costing on average €40,000 per year, equivalent to the annual cost of a nursing home place in Ireland, and a further €20,000 spread over three years, reflecting current per capita expenditure on community dementia care in Ireland [14]. Introducing the budget allocation exercise generated discussion on what services participants needed to cut from their original (first exercise) non-constrained choices in order to meet the new financial constraint, and why decisions were made, with an emphasis on articulating their decision-making calculus.

### Data analysis

The quantitative data from two workshops was compiled from the spread sheet workbooks. The type and amount of services which were allocated for each case type for both the NBC and the BC scenarios were compiled. For the BC scenario, all participant data was included, irrespective of whether the person achieved the target budget level.

Qualitative data from a balance of care study can be used in a number of ways. Previous studies have applied a thematic framework to summarise the key themes emerging from the data [30]. Alternatively, qualitative findings can be used to provide nuance and support or qualification to the quantitative results [29]. This latter approach is taken in this study.

## Results

### No budget constraint

Table 3 shows the average length of the time periods chosen by the participants, and the average recommended provision for an illustrative subset of services for each of these periods under the NBC scenario. The full set of service recommendations for the NBC scenario are shown in the Supplementary Material (Table S1).

**Table 3:** Average length of the time periods and average service levels for an illustrative subset of services under the no-budget-constraint (NBC) scenario

	Post Event	Steady State	Post Event	Steady State	Post Event	Steady State	Post Event	
	**Period 1a**	**Period 1b**	**Period 2a**	**Period 2b**	**Period 3a**	**Period 3b**	**Period 4a**	**Period 4b**
**Event at start of period**	**Diagnosis.**		**Increased care needs. Some degree of supervision necessary.**		**Hospitalised eg minor fall, UTI, pneumonia.**		**Increased care needs and carer strain. Trigger event.**	**Nursing Home.**
Average Length of period in weeks	**7.6**	57.4	**4**	61	**4.8**	60.2	**44**	21
Total No. of Weeks per period		65		65		65		65
Home Help (hours per week)	0.6	1.6	3.4	6.0	12.7	11.8	27.4	0.0
Day Care (days per week)	0.2	0.4	0.6	1.4	2.0	2.4	0.6	0.0
Meals on Wheels (meals per week)	0.0	0.0	0.6	1.8	5.0	5.0	5.8	0.0
Public Health Nurse (visits per period (a)/per year (b))	1.2	4.2	1.0	6.8	3.0	6.2	21.2*	0.0

*Note: Visits per year

For the sub-period following diagnosis (Period 1a) participants recommended a set of services for an average duration of 7.6 weeks under the NBC scenario. This was followed by 57.4 weeks of a steady state service being delivered. Following an increase in care needs (Period 2a) and hospitalisation (Period 3a) a set of enhanced services were recommended for 4.0 and 4.8 weeks respectively. In period 4, participants recommended that the person can be maintained at home with community supports for 44 weeks, and moved to a nursing home for the remainder of the period – 21 weeks. Participants noted trigger events such as carer hospitalisation, behavioural changes swallowing problems, and continence issues for the person with dementia can substantially increase care needs and potentially result in admission to long-stay care.

Under the NBC scenario the recommended level of provision of home help care hours increased from 1.6 hours per week in period 1b to 6.0 hours per week in period 2b, to 12.7 hours in period 3b and 27.4 hours in period 4a. There was little difference between the home help provision for the post hospitalisation event and the steady state period (3a&b). Most of the participants did not recommend day care in the first period (1b). On average, 1.4 days per week were recommended for the second period steady state and 2.4 days in the third period steady state. In period 4a, the final stage of living in the community, only a minority of participants recommended day care, and those who did recommended it for fewer days per week. Meals on wheels and public health nurse visits tended to increase along the continuum towards admission to nursing home care.

Participants emphasised the importance of events and transition periods for resource allocation, particularly following discharge from hospital, one saying: *“that first week at home can make or break a family”*. Another highlighted the role of a link worker between the hospital and community services to ensure a smooth transition so that families are not overwhelmed in the initial period: *“case manager role is lost (at present) – there is no real handover to community colleagues”*.

### Budget constraint

When faced with a budget constraint, participants did not generally alter the time frames (Table 4), but services were reduced across all areas and time periods. Participants in the workshops felt that the budget provided generally reflected the current availability of resources for people with dementia living at home across the various stages. When asked how far away from reality the constrained budget one was, one participant responded *“not too far”.* However, participants found it difficult to stay within this constraint, expanding it to €133,000, 33% above the initial budget made available to them in this exercise to support their choice of services and supports.

**Table 4:** Average length of the time periods and average service levels for an illustrative subset of services under the budget-constraint (BC) scenario

	Post Event	Steady State	Post Event	Steady State	Post Event	Steady State	Post Event	
	**Period 1a**	**Period 1b**	**Period 2a**	**Period 2b**	**Period 3a**	**Period 3b**	**Period 4a**	**Period 4b**
**Event at start of period**	**Diagnosis**		**Increased care needs. Some degree of supervision necessary.**		**Hospitalised eg minor fall, UTI, pneumonia**		**Increased care needs and carer strain. Trigger event.**	**Nursing Home**
Average Length of period in weeks	**7.6**	57.4	**4**	61	**4.8**	60.2	**44**	21
Total No. of Weeks per period		65		65		65		65
Home Help (hours per week)	0.0	1.0	2.6	4.6	5.4	9.0	16.8	0.0
Day Care (days per week)	0.0	0.2	0.4	0.8	0.6	1.2	0.4	0.0
Meals on Wheels (meals per week)	0.0	0.0	0.0	1.6	2.6	3.2	3.6	0.0
Public Health Nurse (visits per period (a)/per year (b))	1.0	1.6	0.6	4.2	1.2	4.6	9.2*	0.0

*Note: Visits per year

Under the BC scenario, the recommended level of provision of home help care hours increased from 1.0 hours per week in period 1b to 4.6 hours per week in period 2b, to 9.0 hours in period 3b and 16.8 hours in period 4a. Home help care hours nearly doubled between the post hospital event and the long steady state period following discharge (3a&b). Day care hours were generally lower than the NBC scenario, only going above one day per week in period 3b. Once again, meals on wheels and public health nurse visits tended to increase along the continuum towards admission to nursing home care. However, levels of provision for both were lower than the NBC scenario, and were more than halved in period 4a for people on the boundary of admission to residential care.

### Cost of care

**Fig. 2:**
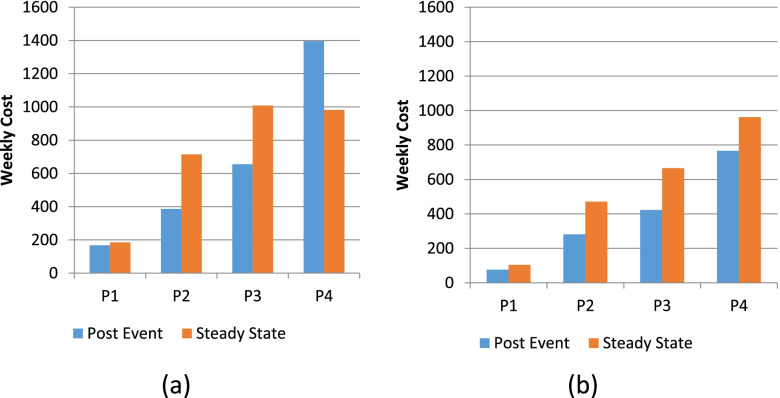
Weekly costs for the NBC (**a**) and BC (**b**) scenarios for each time period

Figure 2a and b show the weekly cost for each of the sub periods under NBC and BC scenarios respectively. Figure 2a shows that for the NBC scenario in periods 1 to 3, fewer weekly resources were allocated in the initial period following the transition event than for the subsequent steady state period. The situation is similar for these stages in the BC scenario, albeit at a lower level of weekly expenditure for all periods. Under the NBC scenario, for period 4a, when the person has high care needs and is living in the community, a higher level of resources are allocated, more than the cost of a nursing home stay (period 4b). Under the BC scenario this situation is reversed with a lower level of weekly expenditure in the period prior to moving into a nursing home. The total cost for the five year period under the NBC scenario is €200,000 on average; this reduces to €133,000 under the BC scenario. Under the BC (NBC) scenarios, participants spent on average 85% (90%) of their budget on community services and 15% (10%) on nursing home services.

The qualitative data indicate that when faced with a budget constraint, participants reduced services at all stages of the continuum, thus seeking to maintain a minimum level of service in earlier and later stages of the disease. As one participant put it: “*I just reduced a bit of everything across the board”.* Moreover, faced with a budget constraint, participants directed scarce resources towards meeting personal care needs rather than psychosocial provision as emphasised by this participant who reported that: “*I cut back on the emotional support*”. Participants recognised that for some people at the end stage of dementia home-based community care was no longer feasible or desirable due to increased nursing needs or carer burden: *“realistically the nursing home probably would have been a factor there because they did not have the support (in the community)”.*

## Discussion

In this study we outline an approach that can be used to identify and prioritise resource allocation and services for people with dementia. The study uses a longitudinal framework to collect data from practitioners on potential services and supports for a representative person with dementia over a five-year period, during which various events and transitions happen. There are many benefits to using this approach including: capturing key transition points in care needs; illustrating the time sequencing of needs among people with dementia; and providing a framework for trading off the costs of various services and supports. Thus, this method can be used to show preferences for different services and supports following an event, such as diagnosis or hospitalisation, for a specified initial period and in the steady state.

The study firstly identifies the set of services that participants would recommend at different stages of dementia if there were no budget constraints. This is useful, as it allows participants to think beyond what is currently typically provided, mainly public health nursing and home care, allowing greater focus on the potential benefits to the person with dementia rather than existing supply. In line with other studies, participants recommended a broad range of health and psychosocial services when there are no budget constraints [21, 29, 30]. However, when making decisions on cutbacks in the budget constrained exercise, participants choose to cut psychosocial services and supports rather than mainstream nursing and home help hours. This is not surprising given the importance of the latter for people with dementia, and the familiarity of participants with this service, but it does indicate the obstacles to the development of a more holistic, person-centred, psychosocial model of care for people with dementia in Ireland.

The results suggest that participants in this type of resource allocation exercise are capable of allocating resources across the different stages of dementia for a wide range of services, under both unconstrained and fixed budget rules. It is interesting that some participants could not keep to the provided budget constraint, exceeding the guide budget level by one third. This was despite agreement that the budget provided was in line with current resources available. That said, even with unconstrained budgets, participants did not always recommend additional spending in response to key transition events. For example, in period 4a, the final stage in the community, only a minority of participants recommended day care, and those that did recommended it for fewer days per week. Overall, however, significant resources were allocated to home care services and supports in an effort to keep people living in their own homes. Participants, faced with an unconstrained budget, recommended spending €400 a week more on home care than alternative nursing home provision.

The qualitative findings are useful in teasing out how and why decisions were made. They confirm the importance of events and subsequent transitions in determining the well-being and quality of life for people with dementia and their carers [23]. The absence of services, or poor quality services, at critical event junctures can lead to poor outcomes for people with dementia [25, 50]. Participants especially highlighted the importance of the quality and timeliness of service delivery after hospitalisation, more so than any increase in the volume of community supports. While post-acute care rehabilitation may be reduced by cognitive impairment, it can still be effective for some people [51].

The junction between care in the community and nursing home care is another important point along the continuum, both economically and socially [52]. Across Europe there is a substantial variation in the availability and utilisation of services and supports at critical transition points along the continuum of care [53]. Our results show that the need for additional resources was recognised by participants at the end stage of the continuum in the NBC scenario, with participants recommending an addition €400 a week more than the cost of nursing home care to provide a good standard of care for people living at home. Previous balance of care studies have explored the service needs of people with dementia living at home on the margin of nursing home care [54], but there has been little analysis of the decision-making process underpinning decision-making or any relationship to previous events across the continuum.

The longitudinal framework introduced in this study facilitates an analysis of resource allocation decision-making across the whole of the dementia continuum – from diagnosis to death. Specifically, the method demonstrates the extent to which stakeholders seek to avoid nursing home placement from a very early stage, in line with peoples strongly stated preferences [55], through enhancing the quantity and quality of community care provision at all stages of the disease. With a cross sectional BoC study the length of time that community care can be maintained is not assessed [54]. The longitudinal approach allows decision-makers to take a longer-term view of the resource allocation process, thereby facilitating a more integrated and holistic approach.

Finally, participants allocated high levels of resources to community services even when budgets were constrained. This is in marked contrast to how resources are allocated in practice with the majority of funding in Ireland going towards residential care [56]. However, the timing of nursing home placement did not change based on the budget condition. This is an area that requires further exploration and was not sufficiently illuminated in the qualitative data. Perhaps participants believe that families will continue to offset any reductions in formal care services with greater levels of informal care, albeit at a personal cost of potentially lower quality of life. Some of the other findings of this study are similar to those found in related studies using a cross section of case types with a budget constraint [21, 29, 30]. Specifically, when faced with budget constraints participants cut back on what they viewed as non-essential psychosocial services.

### Strengths and limitations

Key strengths of this study are the wide array of services that participants could choose from, the inclusion of a budget constraint, the mixed methods design and the longitudinal nature of the exercise. While the small number of participants limits the generalisability of our findings, the main aim of the study is to demonstrate the method and its potential for widening the focus of resource allocation decision-making, especially within the BoC methodology. For example, further work could employ variations in case types by including behavioural and psychological symptoms of dementia [40] and social networks to ascertain whether participants see these factors as influencing or shaping the trajectory care through time. The study used a uniform set of time periods of 65 weeks length; while this is broadly reflective of the average length of time people spend at each stage of dementia, in practice the rate of progression of the condition varies widely. The study also included one co-morbidity to prompt discussion on issues around multi-morbidity. An alternative approach would be to use a multi-morbidity index [57]. In this study the average cost of nursing home care was used. In practice, however, there is significant variation in the cost of nursing home care across the country which could have implications on decisions to support crisis interventions in stage 4 of the analysis, depending on location. Finally, the service choices made by health care professionals may be influenced by their knowledge of the current system and any biases they may have about different services.

## Conclusion

The methodology used in this paper is a valuable complement to cross-sectional BoC studies through its identification of the importance of events, transitions and staging along the dementia care continuum. The methodology facilitates a longer-term focus on resource allocation from initial diagnosis to placement into residential care over a five year period. Diagnosis is the initial stimulus for change in the person’s life and requires an immediate, if low-cost, response from health and social care providers. Over time, there is an increase in both physical and social need as the person moves to a moderate stage of dementia, each requiring a commensurate response from providers. Hospitalisation is also seen as a potential transition event, leading to changes in resource allocation. So too are a range of known, but often unforeseen, trigger events that can accelerate care needs and associated carer burden, including: carer hospitalisation; behavioural changes in the person with dementia; swallowing problems; and continence issues.

This study demonstrated that participants were able to allocate different packages of services and supports for different stages of dementia under different budgetary conditions. Participants found it difficult to adhere to current spending limits when operating under fixed budgets, increasing expenditure by one third over the guide current budget level. The desire of participants to keep people with dementia living in their own home is strong, even in the later stages of dementia, as evident by their recommendation to spend €400 per week more on home care provision compared to the alternative residential care, albeit in the absence of any budget constraints. When faced with cutbacks in the budget constrained exercise, participants chose to cut psychosocial services and supports rather than mainstream provision. While this approach is perhaps understandable, failure to provide psychosocial care for people with dementia will likely impact on their social connectivity, emotional well-being and ability to remain living in their own homes for as long as possible and practicable [58].

## Supplementary Information

**Additional file 1.**

## Data Availability

Supporting data for this paper is available at: 10.5281/zenodo.6385129.
