# Supplementary material for: Exploratory pilot study on resource allocation along the dementia continuum under constrained and unconstrained budget scenarios
Source: BMC Geriatr. 2022 May 18;22:437. doi: 10.1186/s12877-022-03089-1 (PMC9118708; doi:10.1186/s12877-022-03089-1)
Supplement: Supplementary file 1 — Additional file 1. [file 12877_2022_3089_MOESM1_ESM.docx]

**Supplementary material**

**Table S1. Average length of the time periods and average service levels for an illustrative subset of services under the no-budget-constraint (NBC) scenario (Full table).**

|  |  |  |  |  |  |  |  |  |  |
| --- | --- | --- | --- | --- | --- | --- | --- | --- | --- |
|  | **Post Event** | **Steady State** | **Post Event** | **Steady State** | **Post Event** | **Steady State** | **Event** | **Steady State** |  |
|  | **Period 1a** | **Period 1b** | **Period 2a** | **Period 2b** | **Period 3a** | **Period 3b** | **Period 4a** | **Period 4b** |  |
|  |  |  |  |  |  |  |  |  |  |
| **Event at start of period** | **Diagnosis** | | **Increased care needs. Some degree of supervision necessary.** | | **Hospitalised eg minor fall, UTI, pneumonia** | | **Increased care needs and carer strain. Trigger event.** | **Nursing Home** |  |
| Average Length of period in weeks | **7.6** | 57.4 | **4** | 61 | **4.8** | 60.2 | **44** | 21 |  |
| Total No. of Weeks per period |  | 65 |  | 65 |  | 65 |  | 65 |  |
|  |  |  |  |  |  |  |  |  |  |
| **Basics** |  |  |  |  |  |  |  |  |  |
| Home Help (hours per week) | 0.6 | 1.6 | 3.4 | 6.0 | 12.7 | 11.8 | 27.4 | 0.0 |  |
| Day Care (days per week) | 0.2 | 0.4 | 0.6 | 1.4 | 2.0 | 2.4 | 0.6 | 0.0 |  |
| Meals on Wheels (meals per week) | 0.0 | 0.0 | 0.6 | 1.8 | 5.0 | 5.0 | 5.8 | 0.0 |  |
| Public Health Nurse (visits per period (a)/per year (b)) | 1.2 | 4.2 | 1.0 | 6.8 | 3.0 | 6.2 | 21.2 | 0.0 |  |
| **Community Health** |  |  |  |  |  |  |  |  |  |
| Physio (visits per period (a)/per month (b)) | 0 | 0 | 1 | 3 | 5 | 1 | 4 | 0 |  |
| OT (visits per period (a)/per year (b)) | 1 | 1 | 1 | 4 | 2 | 3 | 3 | 1 |  |
| Other Primary Care (Speech and Language/Dietician/Hearing) (visits per period (a)/per year (b)) | 0 | 0 | 0 | 2 | 0 | 1 | 3 | 3 |  |
| Referral to Psychiatry of Old Age Team (package per period (a)/per year (b)) | 0 | 1 | 0 | 2 | 1 | 2 | 2 | 1 |  |
| Day Hospital (visits in period (a)/days per month (b)) | 0 | 1 | 0 | 2 | 3 | 2 | 1 | 0 |  |
| Dementia Advisor (visits per period (a)/per year (b)) | 1 | 2 | 1 | 3 | 1 | 3 | 2 | 0 |  |
| Specialist Dementia/Case Management (visits per period (a)/per year (b)) | 0 | 2 | 0 | 3 | 1 | 3 | 3 | 1 |  |
| Dementia Cognitive Therapies (6 week program in period (a)/6 week programme per year) | 0 | 2 | 0 | 1 | 0 | 2 | 1 | 0 |  |
| Counselling for person with dementia (sessions in period (a)/sessions per year(b)) | 2 | 4 | 1 | 6 | 3 | 4 | 1 | 1 |  |
| Social Worker | 1 | 2 | 0 | 4 | 2 | 4 | 5 | 3 |  |
|  |  |  |  |  |  |  |  |  |  |
| **Respite** |  |  |  |  |  |  |  |  |  |
| In-home Respite/Sitting Service (eg visiting service) | 0 | 0 | 2 | 4 | 8 | 4 | 8 | 0 |  |
| Nursing home based respite (weeks in period (a)/weeks per year (b)) | 0 | 0 | 0 | 4 | 0 | 4 | 6 | 0 |  |
| **Carer Services** |  |  |  |  |  |  |  |  |  |
| Carer Education Programme (sessions in period (a)/6 week programme per year(b)) | 2 | 1 | 1 | 3 | 0 | 1 | 1 | 0 |  |
| Dementia Carer Support Groups (visits in period (a)/sessions per month(b)) | 1 | 1 | 1 | 2 | 3 | 1 | 1 | 1 |  |
| Counselling for family carer (sessions in period (a)/ sessions per year (b)) | 2 | 4 | 1 | 5 | 3 | 6 | 6 | 6 |  |
| **Getting Out** |  |  |  |  |  |  |  |  |  |
| Transport (20 minute round trip per week) | 0 | 1 | 3 | 2 | 2 | 2 | 1 | 0 |  |
| Re-ablement / Dementia support worker (visits per week) | 0 | 0 | 1 | 1 | 1 | 1 | 2 | 0 |  |
| Alzheimer’s Café, Dementia Social Clubs, or other support group for people with dementia (visits in period (a) / visits per month (b)) | 1 | 1 | 1 | 2 | 1 | 1 | 1 | 0 |  |
| Dementia Friendly Activities (visits in period (a)/visits per month (b) ) | 1 | 2 | 1 | 4 | 1 | 3 | 2 | 0 |  |
|  |  |  |  |  |  |  |  |  |  |
